# Unprecedented Insights on Chemical and Biological Significance of *Euphorbia cactus* Growing in Saudi Arabia

**DOI:** 10.3390/plants11050681

**Published:** 2022-03-02

**Authors:** Gadah A. Al-Hamoud, Omer I. Fantoukh, Musarat Amina, Fahd A. Nasr, Nawal M. Al Musayeib, Mohammad Z. Ahmed, Omar M. Noman, Reem E. Al-Sharidah, Fawaz Alasmari, Ali S. Alqahtani

**Affiliations:** 1 Department of Pharmacognosy, College of Pharmacy, King Saud University, Riyadh 11451, Saudi Arabia; mamina@ksu.edu.sa (M.A.); fnasr@ksu.edu.sa (F.A.N.); nalmusayeib@ksu.edu.sa (N.M.A.M.); mahmed4@ksu.edu.sa (M.Z.A.); onoman@ksu.edu.sa (O.M.N.); 438201922@student.ksu.edu.sa (R.E.A.-S.); alalqahtani@ksu.edu.sa (A.S.A.); 2 Department of Pharmacology and Toxicology, College of Pharmacy, King Saud University, Riyadh 11451, Saudi Arabia; ffalasmari@ksu.edu.sa

**Keywords:** *Euphorbia cactus*, phytochemicals, antioxidant, cytotoxicity

## Abstract

*Euphorbia cactus* Ehrenb ex Boiss. is a plant species reported from central Africa and the southern Arabian Peninsula, belonging to the family of Euphorbiaceae. The plant has ethnobotanical values and is well-known for its milky latex, which has been turned into medicine to treat various ailments. To the best of our knowledge, there have been no literature reports available on phytochemical constituents and antiproliferative mechanism of *E. cactus*. In the current study, the phytochemical investigation of *E. cactus* methanolic extract (ECME) resulted in the isolation and characterization of four secondary metabolites, which are reported for the first time from this plant species. In addition, the results of 1,1-diphenyl-2-picrylhydrazyl (DPPH•) and ferrous ion chelating (FIC) assays expressed maximum antioxidant activity by ECME and the isolated phytochemicals. Furthermore, ECME exerted a promising antiproliferative effect against different cancer cell lines, and the A549 lung cancer cells were the most sensitive with an IC_50_ value of 20 µg/mL. The antiproliferative action of ECME in A549 cells was associated with cell accumulation in the G2/M phase and an increase in early and late apoptosis. In addition, RT-PCR and western blot analysis revealed that ECME decreased the anti-apoptotic (Bcl-2) expression, while the expression of pro-apoptotic (Bax) and caspase-3 were increased. This study provides the first insight into the phytochemical constituents and the antiproliferative mechanism of ECME, implying that it could be exploited as a promising natural source for developing new cancer therapies. Further preclinical research is warranted to support the current results.

## 1. Introduction

*Euphorbia* is the third largest genus of flowering plants in the Euphorbiaceae family, with almost 2000 species distributed in tropical and subtropical climate zones. The rich morphological variability and near-cosmopolitan distribution of *Euphorbia* have caught attention worldwide since ancient times [1]. *Euphorbia* species are readily distinguishable by their specialized inflorescences and milky latex [1,2,3]. The plants of this genus are commonly used for ornamental and household purposes [4]. The genus is well known for the chemical diversity of its isoprenoid components [5]. Some plants of this genus are of great importance, and they have been used as traditional folk medicine to treat skin disease, venomous bites, abdominal pain, abdominal distention, trichiasis, as wart removers, and to treat paralysis [6].

Chemically, diterpenoids with various core frameworks such as jatrophanes, ingenanes, lathyranes, myrsinols, and tiglianes are the main components found in *Euphorbia*. Other reported chemical constituents were sesquiterpenoids, cerebrosides, flavonoids, phloracetophenones, steroids, and glycerols [5]. Various pharmacological properties have been reported for the genus *Euphorbia* including antioxidant, antibacterial, antifungal, antiviral, anti-inflammatory, and cytotoxic effects [7,8]. The extract of latex of these plants has shown co-carcinogenic activity due to the presence of diterpene esters (tigliane and ingenane), and it has been banned from commercial uses [9,10]. However, many secondary metabolites found in the latex extract have anticarcinogenic activities [11,12].

*Euphorbia cactus* Ehrenb ex Boiss. (Family; Euphorbiaceae) is a perennial succulent leafless spiny shrub with 3–4 angled dark green branches mottled with radiating yellow streaks. It is widely distributed in central Africa and the southern Arabian Peninsula and reaches up to 3 m high. Fruit capsules are dull red 3-angular with 8–9 × 15–16 mm in size [13]. The extract of *E. cactus* latex showed antileishmanial activity [14], whereas the crude methanolic extract of the whole plant has been reported to exhibit antioxidant, antimicrobial, and anticancer activities [15]. Considering the pharmacological activity of *E. cactus* extracts, different parts of the plants need further investigation. To the best of our knowledge, the plant species has not been extensively explored for its chemical and pharmacological potential. In our continued quest to explore the flora of Saudi Arabia, the current study aimed to investigate the phytochemical, antiproliferative, apoptotic and antioxidant properties of methanolic extract of aerial parts of *E. cactus* (ECME).

## 2. Results and Discussion

### 2.1. Preliminary Phytochemical Screening

The phytochemical study of ECME revealed a broad diversity of phytochemicals. The major phytochemical constituents included phenols, diterpenes, flavonoids, sesquiterpenoids, terpenoids, anthocyanins, tannins, steroids, anthraquinones, carbohydrates, cerebrosides, phloracetophenones, glycerols, and alkaloids were present in the methanol extract (Table 1).

### 2.2. Isolation of Chemical Constituents

**Figure 1:**
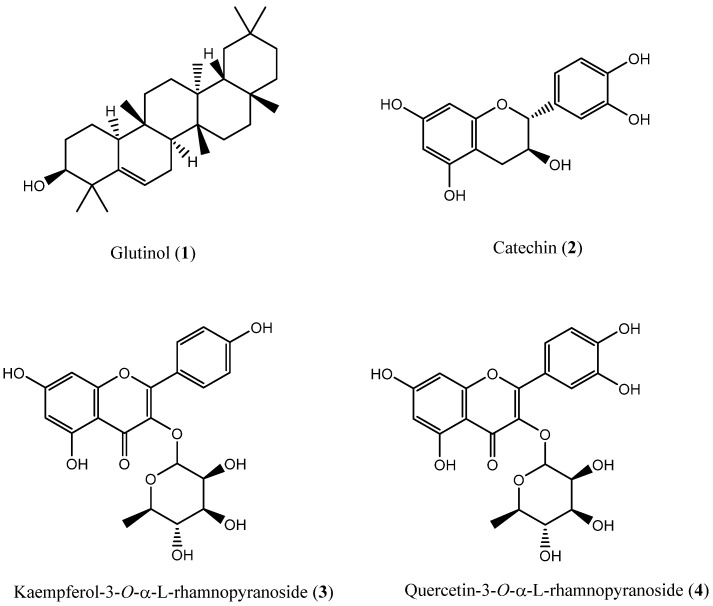
Chemical structures of secondary metabolites isolated from the methanolic extract of aerial parts of *E. cactus* (ECME).

Phytochemical investigation of ECME aerial parts resulted in the isolation and characterization of four secondary metabolites (Figure 1). Their chemical structures were established using NMR, IR, and MS, and by comparison of obtained data with the available literature. Specifically, the obtained compounds were one triterpenoid and three flavonoids, which have been reported for the first time from this plant species and in agreement with the chemotaxonomic profile of the genus *Euphorbia*. Glutinol (**1**) is a rare pentacyclic triterpenoid, and it was isolated previously from the whole plant of *Euphorbia segetalis* [16] and leaves of *Euphorbia ammak* growing in Saudi Arabia [17]. Catechin (**2**) is widely distributed in the plants and was previously reported from *Euphorbia denticulate* [18] and *Euphorbia dracunculoides* [19]. Kaempferol-3-*O*-α-L-rhamnopyranoside (**3**) and quercetin-3-*O*-α-L-rhamnpyranoside (**4**) are well-known active flavonoids and were previously isolated from *Euphorbia davidii* and *Euphorbia sanctae-catharinae* [20,21].

### 2.3. Antioxidant Activity

A free radical is a molecular species containing an unpaired electron and is engaged in bacterial, fungal, and parasitic infections, inflammation, atherosclerosis, lung damage, reperfusion injury, aging, neoplastic, and cardiovascular and autoimmune disorders [22,23]. The results of our study demonstrated that ECME exerted free radical scavenging activity in vitro models including DPPH^•^ and FIC assays.

#### 2.3.1. Free Radical Scavenging Activity (DPPH^•^)

DPPH^•^ free radicals are widely used for investigating the preliminary radical scavenging effect of the plant extract [24]. Scavenging of DPPH^•^ radical is associated with lipid peroxidation inhibition [25]. DPPH^•^ is a substance used to test antioxidant activity [26]. Antioxidants either shift a hydrogen atom or an electron to the DPPH^•^ and neutralize its free radical feature [27]. DPPH^•^ test is based on the ability of stable DPPH^•^ free radical to decolorize in the presence of antioxidants and is considered a reliable procedure for determining the action of radical scavenging [28]. Several studies in the literature have addressed the free radical scavenging activity of different *Euphorbia* species [29,30,31,32]. In the current study, the highest inhibition percentage was observed with ECME (89.75), followed by compound **3** (69.35) and compound **4** (62.21) at 200 µg mL^−1^ (Table 2). Furthermore, it was observed that ECME displayed more pronounced scavenging activity, in contrast to isolated compounds as well as BHT standard (Table 2). This result could be attributed to the synergistic effect of isolated compounds **1**–**4** or other minor components present in the extract.

#### 2.3.2. Ferrous Ion Chelating Assay (FIC)

Iron is an essential metal for life and plays a crucial role in the transport of oxygen, respiration and activity of various enzymes. However, it is a highly reactive metal and catalyzes oxidative changes in proteins, lipids and other components of the cell [33]. The metal chelating capacity of ECME was determined by the ferrous ion ferrozine-Fe^2+^ complex formation. Ferrous ions unite with ferrozine, resulting in a red-colored complex that shows absorbance at 562 nm [34]. Chelating agents forming σ bonds with the metal are considered effective secondary antioxidants as they have the ability to decrease the redox potential and stabilize the metal ion in its oxidized form [34]. The iron-binding ability of ECME and isolated compounds **1**–**4** were measured as a percentage of inhibition, and compound **4** showed the highest potential (62.45%), followed by compound **3** (56.24%) and compound **2** (53.14%) at 3000 µg mL^−1^ concentrations (Table 2). However, ECME and compound **1** exhibited moderate effects at similar concentrations, which was not comparable to that of the EDTA reference standard.

### 2.4. Antiproliferative and Apoptotic Activity of ECME

**Figure 2:**
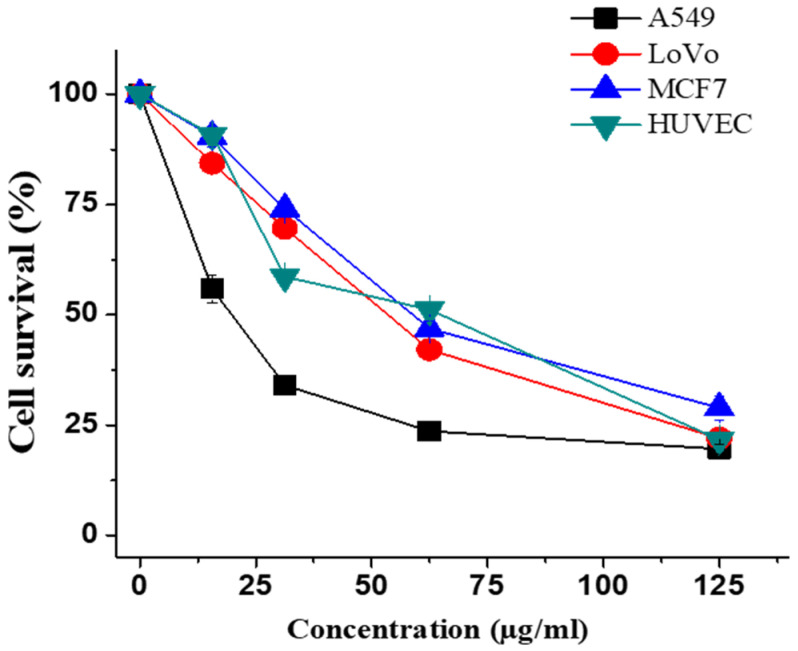
Effect of ECME on the viability of various cancer cells. Cells were treated with increasing concentrations for 48 h, and cell survival was estimated by an MTT assay. The percentage of cells’ survival was determined as compared with the untreated cells.

MTT assay was employed to assess the growth inhibitory effect of ECME on A549, LoVo, MCF-7 cancer cells and HUVEC normal cells to study its antiproliferative effect. We found that ECME reduced the viability of all tested cells in a concentration-dependent manner (Figure 2). The IC_50_ values of ECME on the cancer and normal cells were 20.1 ± 0.5 (A549), 53.2 ± 0.4 (LoVo), 58.80 ± 1.83 (MCF-7), and 65.26 ± 2.80 µg/mL (HUVEC) (Table 3).

In fact, the cytotoxic activity for several species of *Euphorbia* genus, including *E. hirta* [32], *E. formosana* [35], *E. tirucalli* [36], and *E. helioscopia* [37] against various cancer cells have been reported. However, limited studies regarding the cytotoxic activity of *E. cactus* species have been determined. A previous study analyzed the cytotoxic activity of *E. cactus* that grew in Yemen against human bladder carcinoma cell line [38]. Another study found that *E. cactus* methanolic extract exhibited a potent cytotoxic effect against MCF-7 (breast), HepG2 (liver) and PC-3 (prostate) cancer cells with IC_50_ values ranging from 17–27 µg/mL [15]. Here, the IC_50_ values reported were slightly different from the previous study, and this variance could be attributed to differences in the plant parts as well as type of cell lines used in both studies.

Furthermore, compounds **1**–**4** were tested for their antiproliferative activity against the most responsive cells (A549 cells). The results showed that compounds **2**–**4** did not display any cytotoxic activity while glutinol (**1**) exhibited a weak cytotoxic activity (IC_50_ > 100 μg/mL), which were in line with previous studies [21,39]. Thus, a synergistic effect for these compounds may explain the observed antiproliferative activity of the methanol extract. In addition, the observed activity could be attributed to some minor components existed in the extract.

**Figure 3:**
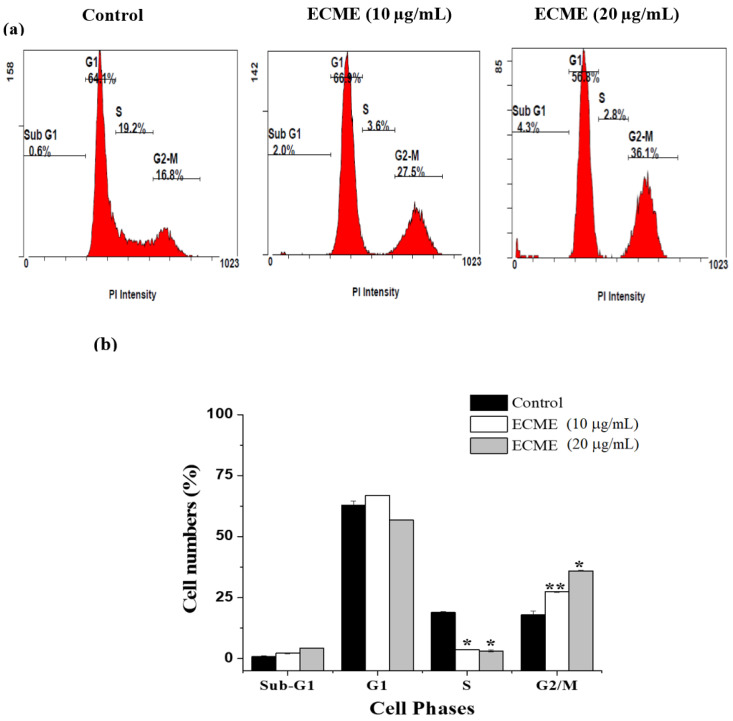
ECME induces G2/M phase cell cycle arrest in A549 cells. (**a**) The histogram shows the distribution of cell phases after treatment with corresponding IC_50_ and its half concentrations (20 and 10 µg/mL) of ECME. (**b**) The values indicate the percentage of cells in the indicated phases of the cell cycle. Significant differences from the control are indicated by * *p* < 0.05; ** *p* < 0.01.

Overall, our results indicated that ECME had a promising growth inhibitory effect on A549 lung cancer cells. Hence, A549 cells were selected for further investigation. Next, flow cytometry was employed to evaluate cell cycle progression to understand the antiproliferative mechanism exerted by ECME. To this end, A549 cells were incubated for 48 h with ECME at the half and IC_50_ concentrations (10 and 20 µg/mL) and stained with propidium iodide (PI). As shown in (Figure 3), ECME increased cell number at G2/M phase to 27.5 ± 0.2 and 36.1 ± 0.2% after 48 h of treatment with 10 and 20 µg/mL respectively compared to untreated cells (16.8 ± 1.4%). This increase was accompanied by decreased cell numbers at the S phase, and this result clearly indicates that ECME caused G2/M cell cycle arrest. In fact, the G2/M phase is required for cell entry into the M phase, and it is also linked to tumor cell resistance [40]. Hence, ECME can be a promising source of agents for cell growth inhibition.

**Figure 4:**
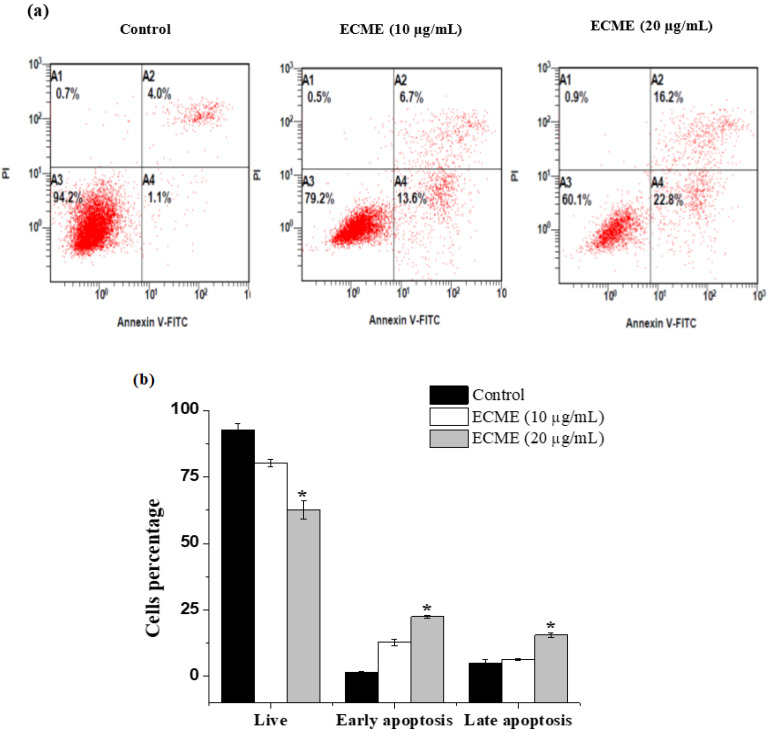
Apoptosis induction in A549 by ECME as detected by Annexin V/PI assay. (**a**) Apoptosis histogram of control cells, and cells treated with 10 and 20 µg/mL. A1: Cells labeled with PI only (necrosis), A2: cells labeled with annexin V and PI (late apoptosis), A3: Viable cells and A4: early apoptotic cells (Annexin V+/PI−) −. (**b**) Apoptosis percentage data from three experiments (mean ± SD, * *p* < 0.05 vs. control group).

Excessive cell proliferation and apoptosis evasion are also among the key characteristics of tumor cells that influence cancer onset and progression [41]. As a result, reducing tumor cell proliferation and inducing apoptosis are considered substantial options for tumor treatment. Therefore, a quantitative assessment of apoptosis was performed by Annexin-FITC/PI staining to investigate whether ECME has mediated cellular apoptosis. As illustrated in (Figure 4), the populations of cells undergoing early and late stages were increased in a concentration-dependent manner after exposure to ECME. The early apoptotic rates in the A549 cells were increased to 13.6 ± 1.1 and 22.8 ± 0.5% while late apoptotic cells were raised to 6.7 ± 0.3 and 16.2 ± 0.8% following treatment ECME at 10 and 20 µg/mL for 48 h, respectively (Figure 4).

**Figure 5:**
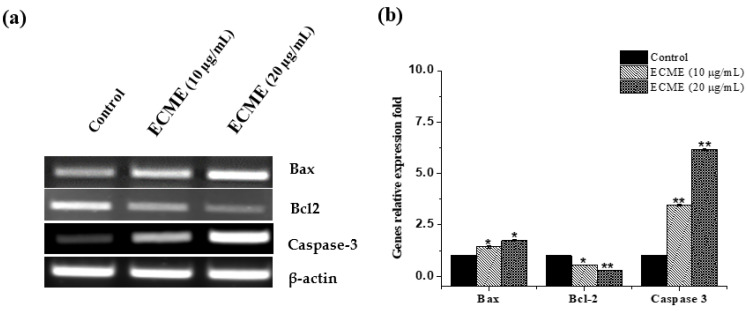
Effects of ECME on the expression levels of Bax, Bcl-2 and caspase 3 genes determined via RT-PCR analysis. A549 cells were exposed to ECME at two effective concentrations of ECME for 48 h, while the negative control was treated with DMSO (0.01%). (**a**) The expression of apoptosis-related genes, including Bax, Bcl-2 and caspase-3 were detected by RT-PCR and β -actin was employed as internal a reference. (**b**) The quantitative level is display in histograms and the average value (n = 3), for the control was set at one. * *p* < 0.05, ** *p* < 0.01 vs. the control group.

**Figure 6:**
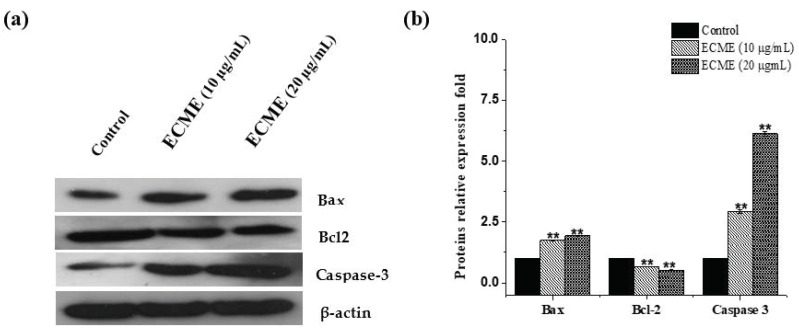
Effect of ECME on protein levels of proapoptotic and antiapoptotic proteins. A549 cells were treated with vehicle (0.01% DMSO) or at two effective concentrations of ECME for 48 h. (**a**) Immunoblot analysis displaying expression levels of Bax, Bcl-2 and Caspase-3. (**b**) Graph depicting the relative intensity of studied proteins versus ECME concentration. The data is the average of three experiments with standard deviation. ** *p* < 0.01 vs. the control group.

It is well known that the anti-apoptotic Bcl-2 and pro-apoptotic Bax proteins play a critical role in the initiation of apoptotic cell death [42]. Other enzymes, mainly caspases, are also among fundamental mediators of apoptosis cell death. Among them, caspase-3, which is widely expressed and known to play a typical role in apoptosis events [43]. Thus, the effects of ECME on cell apoptosis markers, including Bax, Bcl-2 and caspase-3 were determined at gene and protein levels. Following treatment with ECME, our findings revealed that overexpression of Bax and downregulation of Bcl-2 happened simultaneously in A549 cells. A significant increase of caspase 3 was also observed in ECME-treated cells (Figure 5). Additionally, Bax, Bcl-2 and caspase-3 were detected at protein levels in ECME treated cells using western blot. As indicated in (Figure 6), A549 cells exposed to 10 or 20 µg/mL of ECMC display significant (*p* < 0.01) expression levels of Bax and caspase 3 proteins compared to the control. Furthermore, ECME reduced Bcl-2 protein expression with a statistical difference as shown in Figure 6, as compared to untreated cells, which clearly demonstrates the enrollment of these proteins in ECME-induced apoptosis in A549 cells. Since the antiproliferative activity of *E. cactus* species has not been previously investigated at cellular and molecular levels, our study of this species is considered unique to date. On the other hand, the cell cycle arrest and apoptosis induction for several species of *Euphorbia* genus have been documented [7]. Our findings agree with a previously published report which mentioned that *E. hirta* methanol extract has induced apoptotic cell death and G2/M phases arrest in MCF-7 breast cancer cells [44]. In line with our study, the treatment of various cell lines with hexane extracts from three species of *Euphorbia* genus (*E. microciadia*, *E. osyridea* and *E. heteradenia)* resulted in similar apoptotic effects including early and late apoptosis cells populations increment, modulating the ratio of Bax and Bcl-2 expression as well as caspase 3 activation [45].

## 3. Materials and Methods

### 3.1. Plant Material

The aerial parts of *E. cactus* were collected from Fayfa mountains (17°15′01.2″ N, 43°06′40.6″ E) in the southern region of Saudi Arabia in November 2019 and were taxonomically identified by Dr. Ali Mohammed Alzahrani from the Biology Department, Al-Baha University, Saudi Arabia. A voucher specimen (EC-14984) was deposited in the herbarium of the Pharmacognosy Department.

### 3.2. Preparation of Extract

The freshly collected aerial parts of *E. cactus* were thoroughly washed with tap water and rinsed in distilled water before they were cut into small pieces and dried under shade at ambient temperature for 7 days. The dried plant material was finely powdered using a domestic blender and preserved in airtight plastic bags prior to use. The dry powder of the plant sample (300 g) was extracted with methanol solvent (1000 mL) in a Soxhlet apparatus for 72 h at room temperature. The extraction process was repeated three times under similar conditions. All the methanolic extracts were combined, centrifuged, filtered through Whatman No.1 filter paper, and the filtrates were concentrated on a rotary evaporator under reduced pressure at 50 °C to remove the organic solvent. A viscous dark green gummy residue (15.23 g) was obtained and kept at 4 °C before use.

### 3.3. Preliminary Phytochemical Screening

The phytochemical screening of ECME was following previously methods with slight modification [46]. A standard solution was prepared by dissolving 100 mg of extract in 10 mL of methanol. The prepared solution was then evaluated for the existence of different phytochemical components, including phenols, flavonoids, diterpenes, sesquiterpenoids, terpenoids, anthocyanins, tannins, steroids, cerebrosides, anthraquinones, phloracetophenones, glycerols, alkaloids, carbohydrates, and saponins.

### 3.4. Isolation of Chemical Constituents

For the purpose of determining the biological activity and active ingredients, the concentrated methanol extract (10.2 g) of *E. cactus* was diluted with distilled water (100 mL), and the resulting suspension solution was partitioned successively with chloroform (3 × 300 mL), ethyl acetate (3 × 300 mL), and *n*-butanol (3× 300 mL) in the glass separating funnel to obtain 5.7 g of chloroform, 2.2 g of ethyl acetate, 1.4 g of *n*-butanol, and 0.9 g of aqueous fractions, respectively. The chloroform and ethyl acetate fraction showed a similar TLC pattern with numerous phytoconstituents, were combined and taken up for column chromatography over silica gel (230–400 mesh). The elution was performed with a mixture of chloroform and methanol of increasing polarity to obtain six subfractions EC1-EC6. The subfraction EC1 (213 mg) was packed in a glass column (50 × 2 cm) over silica gel (40 g, 60–120 mesh) using as eluent gradient n-hexane: ethyl acetate to yield compound **1** (8.4 mg) as amorphous white solid after recrystallization in MeOH. The subfraction EC2 (325 mg) was chromatographed over SiO_2_ gel column under the same conditions as subfraction EC1 and provided compound **2** (32.3 mg) as a white amorphous powder. The combined subfractions EC3 and EC4 (425 mg) was loaded over silica gel (80 g, 60–120 mesh) in a glass column (50 × 2 cm) using a solvent mixture of CHCl_3_ and MeOH with a gradual increase in polarity yielded compound **3** (20.3 mg). Subfraction EC6 (546 mg) was packed over LH-20 Sephadex column using MeOH as eluent to provide a major fraction, EC-6A (251 mg). The purification of EC-6A (251 mg) was conducted on RP18 (60 g × 2 cm) column and eluted with gradient MeOH: H_2_O to afford compound **4** (6.8 mg) amorphous yellow solid after crystallization in MeOH.

#### Spectral Analysis of Isolated Compounds

*Glutinol* (**1**): White powder; [α]D: +53.85 (c 0.85, CHCl_3_); MS/ESI: *m/z* 426, calculated for C_30_H_50_O, 449 [M+Na]+, IR (KBr) νmax: 2934, 2865, 1643, 3453 cm −1; UV (MeOH) λmax (log ε): 212 (4.36); 1H NMR data (500 MHz, CDCl3) δH (ppm): 5.62 (1H, m, H--6), 3.45 (1H, br s, H--3), 1.21 (3H, s, H--28), 1.15 (3H, s, H--24), 1.12 (3H, s, H--27), 1.08 (3H, s, H--23), 1.03 (3H, s, H--26), 0.99 (3H, s, H--29), 0.97 (3H, s, H--30), 0.83 (3H, s, H--25); 13C NMR (125 MHz, CDCl3) δc (ppm): 141.69 (C--5), 122.16 (C--6), 76.53 (C--3), 49.76 (C--10), 47.50 (C--8), 43.13 (C--18), 40.91 (C--4), 39.37 (C--13), 39.04 (C--22), 37.91 (C--14), 36.10 (C--16), 35.15 (C--19), 33.92 (C--9), 34.69 (C--11), 34.61 (C--29), 33.19 (C--21), 32.50 (C--30), 32.15 (C--15), 32.13 (C--28), 30.44 (C--12), 30.17 (C--17), 29.04 (C--24), 28.34 (C--20), 27.90 (C--2), 25.55 (C--23), 23.72 (C--7), 19.71 (C--26), 18.52 (C--27), 18.30 (C--1), 16.30 (C--25). NMR data were comparable to those reported in the literature was identified as Glutinol [47].

*Catechin* (**2**): Amorphous yellow powder; MS/ESI: *m/z* 290, calculated for C_15_H_14_O_6_, 313 [M+Na]+; IR (KBr) νmax: 3400, 1622, 1523, 1460, 1240, 1130, 1060, 830 cm −1; UV (MeOH) λmax (log ε): 220 (4.25), 290 (2.65) nm; 1H NMR data (500 MHz, CD3OD) δH (ppm): 5.90 (1H, s, H-8), 5.82 (1H, s, H-6), 4.53 (1H, d, H-2), 3.94 (1H, ddd, H-3), 2.82 (4H, dd, H-β), 2.47 (1H, dd, H-4α), 6.81(1H, d, H-6′), 6.73 (1H, d, H-5′), 6.69 (1H, d, H-2′); 13C NMR (125 MHz, CD3OD) δH (ppm): 156.52 (C-7), 156.26 (C-5), 155.59 (C-9), 144.90 (C-4′), 144.90 (C-3′), 130.87 (C-1′), 118.70 (C-6′), 114.72 (C-5′), 113.91 (C-2′), 99.45 (C-10), 94.92 (C-6), 94.13 (C-8), 81.53 (C-2), 67.49 (C-3), 27.21 (C-4). NMR data were comparable to those reported in the literature was identified as Catechin [48].

*Kaempferol-3-O-α-L-rhamnopyranoside* (**3**): Amorphous yellow powder MS/ESI: *m/z* 432 calculated for C_21_H_20_O_10_, 455 [M+Na]+; IR (KBr) νmax: 3550, 2924, 1650, 1610, 1590, 1450 cm−1; UV (MeOH) λmax (log ε): 348 (4.06), 267 (4.04) nm; 1H NMR data (500 MHz, CD3OD) δH (ppm): 7.74 (2H, d, H-2′, H-6′), 6.91 (2H, d, H-3′, H-5′), 6.35 (1H, d, H-8), 6.18 (1H, d, H-6), 5.35 (1H, d, H-1″), 4.19 (1H, dd, H-2″), 3.68 (1H, dd, H-3″), 3.48 (1H, m, H-4″), 3.34 (1H, m, H-5″), 0.89 (3H, d, H-6″); 13C NMR (125 MHz, CDCl3) δc (ppm):178.28 (C-4), 164.80 (C-7), 161.91 (C-5), 160.29 (C-4′), 157.95 (C-2), 157.26 (C-9), 134.86 (C-3), 130.57 (C-2′, 6′), 121.30 (C-1′), 115.20 (C-3′, 5′), 104.52 (C-10), 102.19 (C-1″), 98.57 (C-6), 93.46 (C-8), 71.83 (C-4″), 70.77 (C-3″), 70.71 (C-5″), 70.58 (C-2″), 16.31 (C-6″). NMR data were comparable to those reported in the literature was identified as Kaempferol-3-*O*-α-L- rhamnopyranoside [49].

*Quercetin-3-O-α-L-rhamnopyranoside* (**4**): Amorphous yellow powder; MS/ESI: *m/z* 448 calculated for C_21_H_20_O_11_, 471 [M+Na]+;IR (KBr) νmax: 3190, 2921, 1652, 1356, 1197, 808 cm−1; UV (MeOH) λmax (log ε): 257 (4.25), 356 (4.10) nm; 1H NMR data (500 MHz, CD3OD) δH (ppm): 7.30 (1H, d, H-2′), 7.28 (1H, dd, H-6′), 6.88 (1H, d, H-5′), 6.34 (1H, d, H-8), 6.17 (1H, d, H-6), 5.32 (1H, d, H-1″), 4.19 (1H, dd, H-2″), 3.72 (1H, dd, H-3″), 3.39 (1H, dd, H-5″), 3.31 (1H, dd, H-4″), 0.91 (3H, d, H-6″); 13C NMR (125 MHz, CDCl3) δc (ppm):178.31 (C-4), 164.80 (C-7), 161.89 (C-5), 157.96 (C-9), 157.22 (C-2), 148.50 (C-4′), 145.11 (C-3′), 134.89 (C-3), 121.63 (C-1′), 121.52 (C-6′), 115.58 (C-5′), 115.03 (C-2′), 104.49 (C-10), 102.22 (C-1″), 98.55 (C-6), 93.42 (C-8), 71.91 (C-4″), 70.77 (C-2″), 70.70 (C-3″), 70.57 (C-5″), 16.32 (C-6″). NMR data were comparable to those reported in the literature was identified as Quercetin-3-*O*-α-L-rhamnopyranoside [50].

### 3.5. Antioxidant Activity

The free radical scavenging activity of ECME was assessed by using two different in vitro assays, including DPPH^•^ and FIC assays.

#### 3.5.1. DPPH^•^ Assay

The DPPH^•^ radical-scavenging activity of ECME was evaluated by following the Kirby and Schmidt method [51] with slight modifications. Briefly, 500 µL of ECME at different concentrations (100, 200, 300, 400, and 500 µg/mL) was mixed with 375 µL of methanol (99%), followed by the addition of 125 µL of a DPPH^•^ solution (prepared by 0.2 mM DPPH^•^ in methanol) as a source of free radicals. The reaction mixture was incubated under dark conditions for 30 min at room temperature. After incubation, scavenging capacity was determined spectrophotometrically (UV-VIS T70 Spectrometer, PG Instruments Ltd., Wibtoft, UK) by observing the decrease in absorbance at 517 nm. DPPH^•^ radical displays an absorption band at 517 nm, which completely disappears by an antiradical compound reduction. Butylated hydroxytoluene (BHT) was applied as appositive control. Experiments were performed in triplicates and the inhibition percentage was determined by comparison of the absorbance values of control with the test sample using the following equation:

(1) $$\mathrm{Inhibition}\;\mathrm{percentage}=\frac{Abs_{Control}-Abs_{test\;sample}}{Abs_{control}}\;\times \;100$$

#### 3.5.2. Ferrous Ion Chelating Assay (FIC)

The chelating of ferrous ions by ECME was determined by obeying [52] procedure with slight modifications. Briefly, different concentrations (1000, 2000, 3000, 4000, and 5000 µg/mL) of methanol extract were mixed with 100 μL of 2 mM ferrous sulphate solution and 300 μL of 5 mM ferrozine. The mixed solution was incubated for 10 min at room temperature. After incubation, the absorbance of the solution was recorded at 562 nm. All the tests were carried out in triplicates and the standard used was ethylene diamine tetra acetate (EDTA). The inhibition percentage was determined using the Equation (1).

### 3.6. Cytotoxicity Assay

The cytotoxic activity of ECME against A549 (lung), LoVo (colon), MCF-7 (breast) cancer cells and normal HUVEC cell line was determined by MTT assay as previously described [53]. Cells were seeded in 96 well plates with a count of 5 × 10^4^ cells per well. After 24 h of incubation, cells were treated with the extract at different concentrations (125, 62.5, 31.25 and 15.625 μg/mL), doxorubicin as a positive control or dimethyl sulfoxide (0.01% DMSO) as a vehicle negative control and incubated for 48 h. After incubation, MTT (5 mg/mL, 10 µL) was added per well and further incubated for 4 h. Thereafter, the medium was removed, and the purple formazan crystals were dissolved in isopropanol containing 1N HCl. All samples were treated in triplicate and the absorbance was measured at 570 nm with a multi-plate reader (Bio-Tek, Elx-800, Winooski, VT, USA). Cell viability was calculated as (%) = (O.D of the treated sample)/(O.D of the untreated sample) × 100. The IC_50_ (concentration of the extract that inhibits 50% of cell growth) was generated from the concentration-response curve.

### 3.7. Cell Cycle Analysis

Cell cycle assay was carried out according to the protocol reported by [54]. In brief, A549 cells were plated in 6-well culture plates. After 24 h of incubation, cells were exposed to corresponding IC_50_ and its half concentrations (20 and 10 µg/mL) of ECME or DMSO as a control. At the endpoint of treatment (48 h), the cells were detached, harvested by centrifugation, washed twice with ice-cold PBS and fixed in ice-cold absolute ethanol for 4 h at 4 °C. Thereafter, the cell pellet was resuspended and incubated with a 0.5 mL propidium iodide (PI) staining solution (50 µg/mL PI and 100 µg/mL RNase A) for 30 min in the dark. The cell cycle stages were analyzed using a FACS flow cytometer (Cytomics FC 500; Beckman Coulter, Brea, CA, USA). CXP software v.3.0 was used for data collection and analysis.

### 3.8. Annexin V-FITC/PI Apoptosis Detection

The protocol was followed according to the manufacturer’s instructions (Biolegend, USA). In brief, at 48 h after treatment, floating cells and adherent cells were collected, and the pelleted cells were washed with PBS. Thereafter, the pelleted cells were resuspended in Annexin binding buffer (100 µL) and transferred to cytometer tubes. Cells staining was performed by the addition of 5 µL from both dyes (5 µL of FITC Annexin V and 5 µL propidium iodide) and incubated (10–15 min) in the dark. This was followed by the addition of 0.4 mL of incubation buffer, and the cells were analyzed immediately on FACS flow cytometer (Cytomics FC 500; Beckman Coulter, Brea, CA, USA).

### 3.9. RNA Extraction and RT-PCR

A549 cells were cultured in 6-well plates at 2.5 × 10^5^ cells per 2 mL for each well. On the next day, the media were changed, and the cells were either treated with the vehicle (0.1% DMSO) or plant extract (dissolved in DMSO) at 10 and 20 μg/mL concentrations. Total RNA from the vehicle and treated cells were prepared using Trizol reagent as described by the manufacturer (Invitrogen, Thermo Fisher Scientific, lnc., Waltham, MA, USA). An equal amount of RNA (1 µg) was used to synthesize cDNA using a cDNA synthesis kit (Invitrogen, Thermo Fisher Scientific, lnc., Waltham, MA, USA) according to the manufacturer’s guideline and was used as a template for RT-PCR. A semiquantitative PCR was carried out to determine the expression level of caspase-3, Bax, and Bcl-2, while β-Actin was used as an internal control. The final volume (20 μL) of the RT-PCR mixture, which consist of 2 μL of cDNA, 4 μL of 5X FIRE pol Master mix (Solis Bio Dyne, Tartu, Estonia), and 10 pmol of each complementary primer specific for their respective genes were used. The sequences of specific primers were as follows: Bax 5′-TTTGCTTCAGGGTTTCATCC-3′, and R: 5′-ATCCTCTGCAGCTCCATGTT-3′; Bcl-2 F: 5′-TGATGCCTTCTGTGAAGCAC-3′ and R: 5′-ACAGGCGGAGCTTCTTGTAA-3′; caspase-3: F: 5′-TGGAATTGATGCGTGATGTT-3′ and R: 5′-GGCAGGCCTGAATAATGAAA-3′ and β-actin: F: 5′-CATCGTGATGGACTCTGGTG-3′ and R: 5′-TTTGATGTCACGCACGATTT-3′. The sample was initially denatured at 95 °C for 5 min and amplified using 32 cycles of denaturation at 95 °C for 30 s, annealing at 55 °C for 60 s, and elongation at 72 °C for 60 s. followed by final elongation at 72 °C for 5 min. The final amplification products of 20 μL were run on 1.2% of agarose gel ethidium that was bromide-stained, and the gel picture was taken on LICOR gel doc.

### 3.10. Western Blot

A549 cells were treated either with the vehicle (0.1% DMSO) or plant extract (dissolved in DMSO) at 10 and 20 μg/mL concentrations for 24 h. After 24 h of treatment, the cells were washed twice with 1x PBS, and then cells were lysed by resuspending in 150 μL of lysis buffer (20 mM Tris (pH 7.5), 150 mM NaCl, 1 mM sodium EDTA, 1 mM ethylene glycol-bis (β-aminoethyl ether)-*N*,*N*,*N*′,*N*′-tetraacetic acid, 1% Triton X-100, 1 µg/mL leupeptin, and 100 µM phenylmethylsulfonyl fluoride). The cell lysates were centrifuged, and total protein content was determined using Bio-Rad reagent. Proteins were transferred onto the nitrocellulose membrane following the electrophoresis by the wet transfer method using Bio-Rad electrotransfer apparatus (Bio-Rad Laboratories, CA, USA). The membranes were then blocked with 5% BSA (in Tris buffer saline containing 0.1% Tween 20) by incubating it for 2 h at room temperature, The membranes were washed 3X with TBST and then incubated with the desired primary antibodies on the rocker at 4 °C for overnight. On the next day, membranes were washed 3X with TBST and then incubated with horseradish peroxidase (HRP) conjugated secondary antibodies (at room temperature for 1 h). The membranes were washed 3X, and the ECL (Thermo Scientific) solution was used to detect the signal by exposing it to an X-ray film.

### 3.11. Statistical Analysis

OriginPro 8.5 software was used to conduct statistical analysis, display data graphically and IC_50_ values calculation. The statistical differences between control and treated groups were analyzed using Student’s paired t-test. Data was presented as mean ± SD of three experimental observations. Statistical significance was defined as ** *p*  <  0.01, * *p*  <  0.05.

## 4. Conclusions

Phytochemical investigation of ECME resulted in one triterpenoid along with three flavonoids which have been reported from *E. cactus* for the first time. Furthermore, ECME was found to display an antiproliferative activity towards various cancer cells, especially the A549 lung cancer cells, possibly via induction of apoptosis and G2/M cell cycle phase arrest. Apoptosis mediating by ECME was potentially through modulating of Bax, Bcl-2, and caspase-3 proteins. These findings are the primary insights to demonstrate the antiproliferative mechanism of *E. cactus,* suggesting that it might be a promising natural source for developing novel therapeutics against cancer. However, further preclinical studies should be conducted to support the current results.

## Figures and Tables

**Table 1:** Qualitative screening of phytoconstituents present in the methanolic extract of aerial parts of *E. cactus* (ECME).

Phytochemicals	ECME
Phenols	+++
Flavonoids	++
Diterpenes	+++
Sesquiterpenoids	++
Terpenoids	++
Anthocyanins	++
Tannins	++
Steroids	++
Cerebrosides	+
Anthraquinones	++
Phloracetophenones	+
Glycerols	+
Alkaloids	+
Carbohydrates	++
Saponins	-

+++ (Pesent in excess), ++ (Present significantly), + (present in traces), - (absent)

**Table 2:** Free radical scavenging (DPPH^•^) and ferrous ion chelating (FIC) activity of ECME and isolated phytochemicals.

Sample	DPPH^•^ Radical Scavenging		Ferrous Ion Chelating Activity	
	Concentration (µg mL^−1^)	Inhibition (%)	Concentration (µg mL^−1^)	Inhibition (%)
ECME	200	89.75 ± 0.35	3000	36.12 ± 0.45
Compound 1	200	52.34 ± 0.26	3000	41.23 ± 0.26
Compound 2	200	49.12 ± 0.34	3000	53.14 ± 0.22
Compound 3	200	69.35 ± 0.24	3000	56.24 ± 0.36
Compound 4	200	62.21 ± 0.14	3000	62.45 ± 0.42
BHT	200	41.16 ± 0.36	-	-
EDTA	-	-	3000	95.58 ± 0.45

BHT and ETDA were used as reference standards. Values were measured in triplicates and represented as mean ± SD.

**Table 3:** IC_50_ of ECME against cancer (A549, LoVo, and MCF-7) and normal (HUVEC) cell lines as measured by MTT assay. Values were measured in triplicates and represented as mean ± SD and presented as µg/mL.

Cell lines and IC_50_ (μg/mL)				
	A549	LoVo	MCF-7	HUVEC
ECME	20.1 ± 0.5	53.2 ± 0.4	58.80 ± 1.83	65.26 ± 2.80
Doxorubicin	1.20 ± 0.02	1.30 ± 0.05	1.40 ± 0.02	4.1 ± 0.2

## Data Availability

All data generated or analyzed in the current study are included in this article.

## Abbreviations

ECME	*E. cactus* methanolic extract.
MeOH	Methanol.
DMSO	Dimethyl sulfoxide.
DPPH	2,2-diphenyl-1-picrylhydrazyl.
FIC	ferrous ion chelating.
BHT	Butylated hydroxytoluene.
ETDA	Ethylenediaminetetraacetate.
MTT	3-[4,5-dimethylthiazole-2-yl]-2,5-diphenyltetrazolium bromide.
MCF7	Michigan Cancer Foundation-7.
V-FITC	V-fluorescein Isothiocyanate.
RT-PCR	Reverse transcription polymerase chain reaction.
